# Using Range-Wide Abundance Modeling to Identify Key Conservation Areas for the Micro-Endemic Bolson Tortoise (*Gopherus flavomarginatus*)

**DOI:** 10.1371/journal.pone.0131452

**Published:** 2015-06-26

**Authors:** Cinthya A. Ureña-Aranda, Octavio Rojas-Soto, Enrique Martínez-Meyer, Carlos Yáñez-Arenas, Rosario Landgrave Ramírez, Alejandro Espinosa de los Monteros

**Affiliations:** 1 División de Posgrado, Instituto de Ecología, A. C., El Haya, Xalapa, Veracruz, México; 2 Biología Evolutiva, Instituto de Ecología, A. C., El Haya, Xalapa, Veracruz, México; 3 Departamento de Zoología, Instituto de Biología, Universidad Nacional Autónoma de México, Ciudad Universitaria, Ciudad de México, México; 4 Ecología Funcional, Instituto de Ecología, A. C., El Haya, Xalapa, Veracruz, México; Federal University of Goiás, BRAZIL

## Abstract

A widespread biogeographic pattern in nature is that population abundance is not uniform across the geographic range of species: most occurrence sites have relatively low numbers, whereas a few places contain orders of magnitude more individuals. The Bolson tortoise *Gopherus flavomarginatus* is endemic to a small region of the Chihuahuan Desert in Mexico, where habitat deterioration threatens this species with extinction. In this study we combined field burrows counts and the approach for modeling species abundance based on calculating the distance to the niche centroid to obtain range-wide abundance estimates. For the Bolson tortoise, we found a robust, negative relationship between observed burrows abundance and distance to the niche centroid, with a predictive capacity of 71%. Based on these results we identified four priority areas for the conservation of this microendemic and threatened tortoise. We conclude that this approach may be a useful approximation for identifying key areas for sampling and conservation efforts in elusive and rare species.

## Introduction

Spatial variation in abundance across species’ geographic ranges has been a topic of interest for decades (e.g., Brown [1]; Brown et al. [2]; Gaston et al. [3]; Guo et al. [4]; Pearce & Ferrier [5]). A widespread pattern across taxonomic groups is that in most occurrence sites within species’ ranges population numbers are generally low, whereas a few sites have orders of magnitude more individuals [6]. Abundance does not often follow a spatial radial pattern, in which maximum abundance is held towards the geographic center of species’ range and decreases towards the edges [7,8]. Spatial patterns of abundance in nature are more complex than this simple rule and several factors seem to have an influence [9].

First, the processes driving the distribution-abundance relationship operate at different spatial and temporal scales (e.g., local resource availability, environmental suitability of the landscape, dispersal capacity of the species) [10]. Second, spatial patterns are strongly autocorrelated (i.e., nearby sites tend to have more similar abundances than sites that are far apart [1]); therefore, the stronger the spatial autocorrelation, the smaller the changes in population size from site to site across the species’ range [11]. According to Brown and collaborators [2], population abundance is determined by two main factors: (i) the degree to which local conditions fulfill the niche requirements of species, and (ii) the interactions between abiotic and biotic variables. Therefore, spatial variation in abundance depends on the number and nature of niche variables and the way these vary across space.

Abundance is a key parameter for conservation purposes, since it is frequently used as one of the criteria for deciding whether a species is rare or common: for rare species there are few individuals per sample, and thus low absolute variation among samples [2,12,13]. In spite of its importance, abundance is seldom measured across the geographic range of the species because it is time-consuming and requires a great deal of effort, and resources. Species abundance is usually estimated based on data from a handful of sites [14,15].

Methods for estimating the spatial distribution of abundance include the fractal method [16] and the negative binomial distribution [17]. A recent study [18] has proposed an alternative approach, namely the distance to the niche centroid (DNC) method. This is based on Hutchinson’s [19] theory of the multidimensional ecological niche and a further theoretical development, which suggests that abundance, is determined by the internal structure of the niche. The optimal conditions (i.e., where birth rate is maximal and death rate is minimal and thus abundance is highest) occur toward the geometric centroid of the niche in ecological space, and abundance decreases with distance from the centroid [20]. This ecological centrality principle would thus manifest in different geographic patterns across the landscape, depending on the eco-spatial structure [18]. The DNC method fits a curve for the relationship between known abundance samples across the species’ geographic range and the distance to the ecological niche centroid to make range-wide estimates of the species’ abundance. It has proven robust in different geographic contexts and at different scales [21].

**Fig 1:**
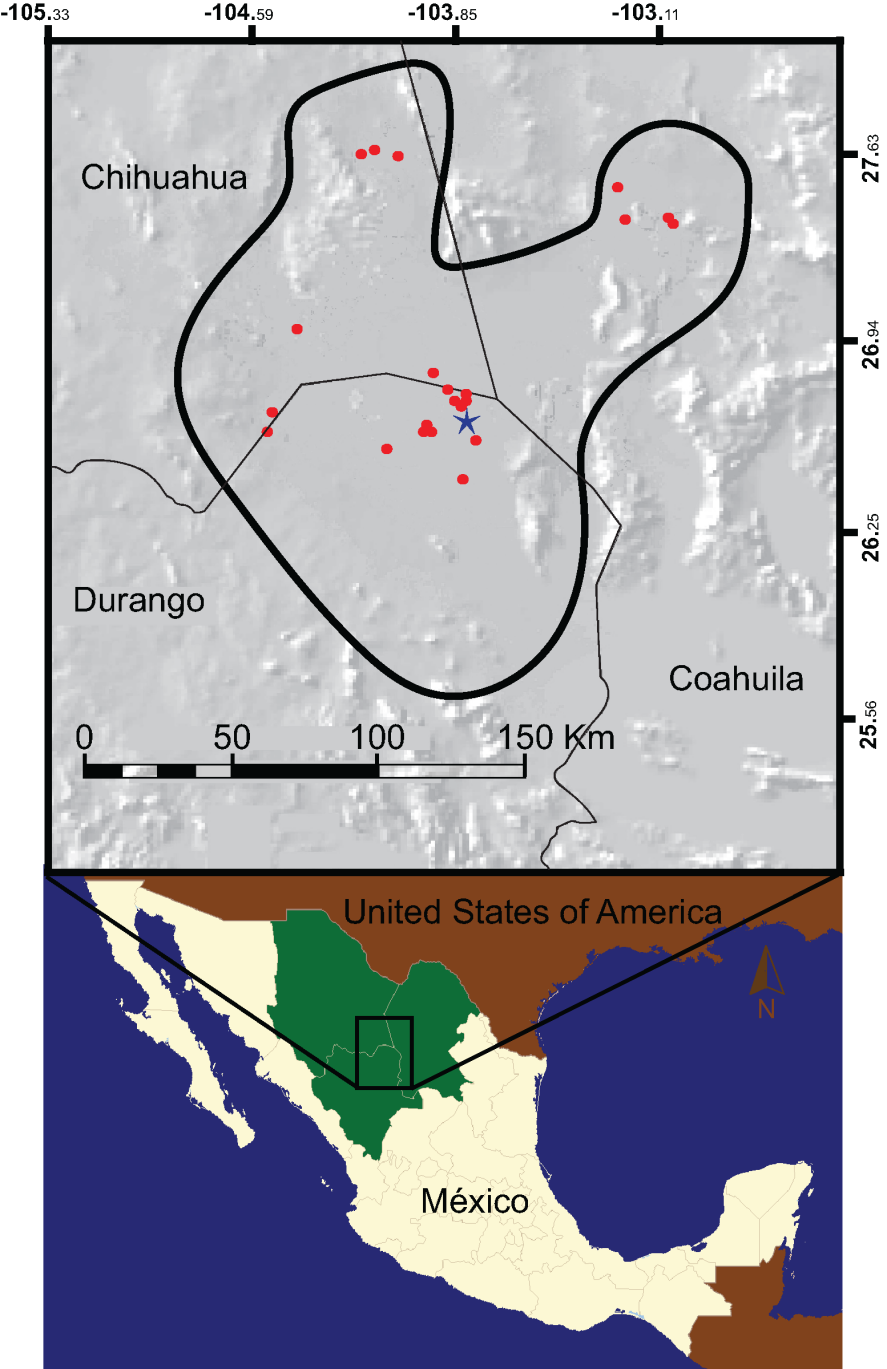
Geographic distribution of *Gopherus flavomarginatus*. The thick black line delineates the polygon of the Mapimí Biosphere Reserve, whereas the state borders are mark by the thin black lines. Red dots pinpoint the sites where the abundance data for the Bolson tortoises was recorded. The star indicates the location of the Mapimí field station.

*Gopherus flavomarginatus* (the Bolson tortoise) is endemic to a portion of the Mapimí Basin, in the Chihuahuan Desert, Mexico (Fig 1). Widespread until the Pleistocene when it ranged from southern USA to central Mexico [22], several factors have brought the Bolson tortoise to the verge of extinction: climate changes in the Pleistocene-Holocene transition, recent anthropogenic activities, including habitat destruction due to overgrazing and agriculture, overexploitation for wildlife trade [23–26], and low genetic variation [27].

Limiting factors in the distribution of the Bolson tortoise include very specific habitat requirements, such as soft slopes and the presence of dry lake beds for nesting [28]. The only previous study in which abundance was estimated reported a mean density of 10 tortoises/Km^2^, and an area of occupancy covering 6,090 Km^2^ [29]. Based on field surveys and niche modeling, in this study we estimated the geographic and ecological distribution of the Bolson tortoise, as well as its range-wide abundance with the DNC method. These data helped us to identify critical areas for concentrating future fieldwork and conservation efforts.

## Materials and Methods

### Ethics statement

The current study was carried out across the whole distribution of *Gopherus flavomarginatus*, including the Mapimí Biosphere Reserve. Nonetheless, special permits were not required. This study did not involve any sort of tortoise or habitat manipulation. In addition to the Mapimí Biosphere Reserve we obtained access to private properties Rancho Americanos, Rancho La Mena, Rancho Cerros Emilio, Rancho La Parva, Rancho Los Remedios, Rancho San Miguel; and some Ejidos, including Las Flores, Lagunetas, Emiliano Zapata, and Ejido Vicente Guerrero. If needed, the authors can be contacted to provide further details from the respective owners or legal representatives.

### Study area

The current known distribution of *Gopherus flavomarginatus* occupies the central part of the Chihuahuan Desert, in the Bolsón de Mapimí, which covers parts of the states of Chihuahua, Coahuila and Durango (Fig 1). The Bolsón de Mapimí is an endoreic basin composed of a series of small sub-basins intermixed with valleys, but is generally flat with a mean altitude of 1,150 m.a.s.l. Climate is of the type “continental tropical arid” with a mean annual temperature of 20.8°C and an annual precipitation of 264.2 mm [30]. The main vegetation type is desert shrubland [31] and the Bolsón has the richest herpetofauna of the whole Chihuahuan Desert with several endemic species, including the Bolson tortoise [32].

### Field surveys

We gathered two types of the tortoise presence information: occurrence and abundance data. Occurrence data were drawn from electronic databases (www.gbif.org and http://www.conabio.gob.mx/remib/doctos/remib_esp.html) and the literature [29,33]. Based on that information we went to the field to record the presence of the species across its distribution range from April 2008 to May 2010. The occurrence data were used to build a niche model for the Bolson tortoise via Mahalanobis distances (see below). Whereas, the abundance data were gathered in 22 independent ~1 km^2^ plots randomly distributed across the distribution range of the species. In each plot we counted all active and inactive adult burrows, excluding all abandoned or destroyed ones. Active burrows were identified by the presence of tortoise footprints and food leftovers (e.g., grass, leaves), whereas other animals such as owls and snakes usually inhabit the abandoned ones. Abundance data were used to calibrate and validate our abundance model (see below).

### Ecological Niche Modeling and distances to the centroid

We assembled a data matrix with the 19 bioclimate variables from the WorldClim database [34], and three topographic variables from the Hydro 1k database [35] (Table 1). All variables were under the geographic coordinate system (WGS84 Datum), and pixel size was 30 arc-seconds (~1 km^2^).

**Table 1:** Climate and topographic variables used for inferring the ecological niche and abundance models of the Bolson tortoise.

Name	Variable
BIO 01	Mean Annual Temperature
BIO 02	Diurnal Temperature Range
BIO 03	Isothermality
BIO 04	Temperature Seasonality
BIO 05	Max Temperature of the Warmest Month
BIO 06	Min Temperature of the Coldest Month
BIO 07	Temperature Annual Range
BIO 08	Mean Temperature of the Wettest Quarter
BIO 09	Mean Temperature of the Driest Quarter
BIO 10	Mean Temperature of the Warmest Quarter
BIO 11	Mean Temperature of the Coldest Quarter
BIO 12	Annual Precipitation
BIO 13	Precipitation of the Wettest Month
BIO 14	Precipitation of the Driest Month
BIO 15	Precipitation Seasonality
BIO 16	Precipitation Wettest
BIO 17	Precipitation of the Driest Quarter
BIO 18	Precipitation of the Warmest Quarter
BIO 19	Precipitation of the Coldest Month
CTI	Compound Topographic Index (or Wettest Index)
ALT	Altitude
SLOPE	Slope

Currently, there are many algorithms to produce niche-based distribution models, and their performance capacity varies depending on the type, amount and bias of the biological data [36]. Due to the collinearity observed in the environmental variables, for this study we implemented the Mahalanobis Distance method. Fieldwork yielded a total of 241 confirmed presence points. These points were used to infer the species ecological niche by obtaining the particular values for each environmental variable (Table 1). Then, these values were used to calculate a multidimensional environmental mean (i.e., the niche centroid), then, Mahalanobis distances were calculated from the niche centroid to each occurrence point. We considered as the potential distribution of the Bolson tortoise in *environmental space* the climate envelope generated around the 241 occurrence points with a radius equal to the distance observed from the niche centroid to the farthest of such points. Finally, Mahalanobis distances were calculated for all pixels in the study area where the potential distribution of the species *in geographic space* thus encompassed all pixels with distance values equal to or lower than the maximum distance to the occurrence points.

### Relationship between the distance to the centroid and abundance, and the generation of the abundance map

We performed regression analyses between the distance to the centroid and the observed abundance of the Bolson tortoise to find the best fit using the Statistical Package SPSS Ver. 19 (IBM). Then, using the best-fit model we generated the estimated abundance map of the Bolson tortoise across its entire potential distribution range in ArcGis v.10.0 (ESRI, Redlands, CA, USA).

### Abundance Model validation and Uncertainty Map

The predictive capacity of the abundance model (i.e., best-fit model) was assessed applying a regression procedure using a random 70/30 data split for training/validation. We re-estimated the best-fit model using the 70% fraction of the data, and the resulting function was used to predict the abundance of the remaining 30% points. Then, we performed a simple linear regression between the expected and the observed abundance of the 30% data fraction. The R^2^ of this second regression is proportional to the predictive power of the inferred model. We repeated this procedure ten times to observe the standard deviation of R^2^.

Alternatively, we applied a bootstrap procedure consisting of generating 1000 estimates of abundance using a random 65/35 data split for training/validation. We calculated the 95% regression confidence intervals for the iterations, and counted the number of points that fell within them. In this way, we obtained a percentage that reflects the predictive capacity of the model. Finally, we generated an uncertainty map using the confidence intervals [21]. The bootstrap was implemented in R-v.2.15.1 software (R Development Core Team).

## Results

**Fig 2:**
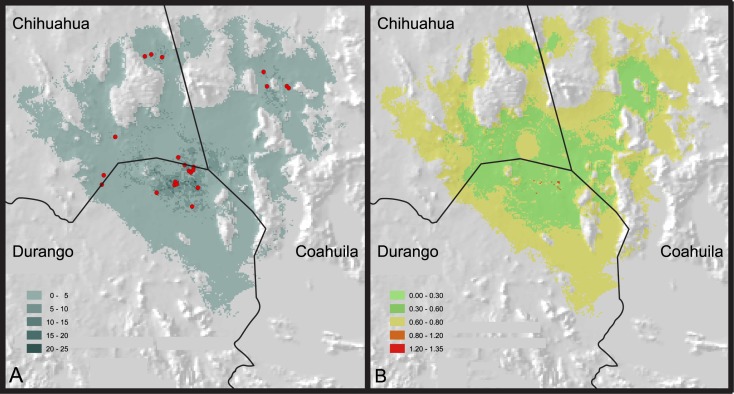
Maps showing the potential distribution and abundance of *Gopherus flavomarginatus*. (A) Inferred abundance for the Bolson tortoise (burrows/Km^2^); red dots pinpoint the sites where the abundance data for the Bolson tortoises was recorded. (B) Standard deviation of abundance.

The potential distribution of the Bolson tortoise, inferred with the Mahalanobis distance method, match well with its known distribution [29] (Fig 2A). Most of the predicted area corresponds to flat lands. The map, however, include a few sites with steep slopes from which the species is known to be absent. Those areas, therefore, were removed from the potential distribution.

**Fig 3:**
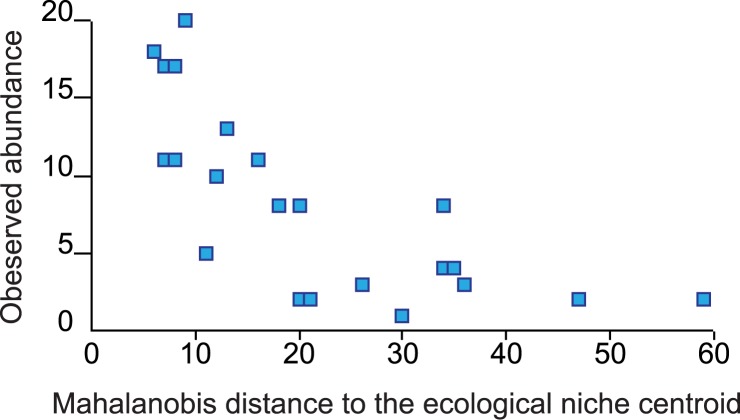
Relationship between the observed abundance (burrows/Km^2^) of *Gopherus flavomarginatus* and the distance to the species´ ecological niche centroid.

All the regression functions that we explored found a significant relationship between the observed abundance and the distance to the ecological niche centroid (Table 2). The best-fit model, nonetheless, was the Inverse Regression explaining more of the variation in the relationship between the Mahalanobis distance to the centroid and the abundance (adjusted R^2^ = 0.681, P < 0.001). As expected, the observed abundance drops drastically when the environmental conditions depart from those found nearby the ecological niche centroid. Our field data showed that the abundance value drops nearly two thirds once the Mahalanobis distance to the niche centroid increase beyond 20 units (Fig 3).

**Table 2:** Goodness-of-fit of the regression models between Mahalanobis distance to the ENC and burrow abundance of *Gopherus flavomarginatus*.

Model	regular R^2^	adjusted R^2^	P-value
Inverse	0.696	0.681	< 0.001
Logaritmic	0.677	0.660	< 0.001
Cubic	0.707	0.658	< 0.001
Quadratic	0.680	0.647	< 0.001
Power	0.628	0.609	< 0.001
Growth	0.545	0.522	< 0.001
Exponential	0.545	0.522	< 0.001
Logistic	0.545	0.522	< 0.001
Lineal	0.540	0.517	< 0.001
SAR	0.685	125.923^1^	< 0.001

^1^ Results inferred from Akaike Information Criterion (AIC)

The highest estimated abundance of the Bolson tortoise is recovered mainly towards the center of the potential distribution area predicted by the Mahalanobis distance method (Fig 2A). This putative rich section is encompassed within the polygon of the Mapimí Biosphere Reserve. However, several other potentially rich areas are predicted outside of officially protected lands. Two of those areas are located in remote isolated regions. One is north of Sierra del Diablo Mountains, in the state of Chihuahua, whereas the other is east of Sierra Mojada Mountains, in the state of Coahuila. Nonetheless, other unprotected areas with high abundance are close to populated zones and are accessible by dirt roads.

According to the validation procedure, the predictive capacity of the best-fit model averaged an R^2^ of 0.712 (p = 0.023, standard deviation = 0.127; Table 3). Abundance estimates are within reasonable ranges (1 to 20 burrows/km^2^), and coincide with our observed field data (Table 4). The uncertainty map, generated with the bootstrap procedure, shows that the inferred abundance has a standard deviation between 0 and 1.35 (Fig 2B).

**Table 3:** Validation for the Inverse Regression Model between Mahalanobis distance to the ENC and burrow abundance.

	Trial	R	R^2^	P-value
	1	0.779	0.638	0.031
	2	0.843	0.653	0.017
	3	0.812	0.660	0.026
	4	0.669	0.447	0.101
	5	0.886	0.785	0.008
	6	0.887	0.787	0.008
	7	0.923	0.852	0.003
	8	0.927	0.858	0.003
	9	0.805	0.648	0.029
	10	0.891	0.794	0.007
Mean		0.844	0.712	0.023
Standard deviation		0.078	0.127	0.029
Minimum		0.669	0.447	0.003
Maximum		0.927	0.858	0.101

**Table 4:** Recorded abundance of *Gopherus flavomarginatus* expressed as the number of burrows/Km^2^.

Sampling Locality	District	Abundance
Americanos	I: Los Americanos, Coahuila	8
Las Flores	I: Los Americanos, Coahuila	2
Lagunetas	I: Los Americanos, Coahuila	4
La Mena	I: Los Americanos, Coahuila	3
Cerros Emilio	II: Sierra del Diablo, Chihuahua	8
Ejido Emiliano Zapata	II: Sierra del Diablo, Chihuahua	2
La Parva	II: Sierra del Diablo, Chihuahua	2
Los Remedios	VI: Sierra de los Remedios, Chihuahua	1
San Miguel	III: Rancho Diana, Chihuahua	4
Ejido Vicente Guerrero	III: Rancho Diana, Chihuahua	2
El Pujo	V: MCR^1^, Durango-Coahuila-Chihuahua^2^	11
Tortugas	V: MCR^1^, Durango-Coahuila-Chihuahua^2^	10
La Flor (Brecha)	V: MCR^1^, Durango-Coahuila-Chihuahua^2^	13
Las Lolas	V: MCR^1^, Durango-Coahuila-Chihuahua^2^	18
Cajones	V: MCR^1^, Durango-Coahuila-Chihuahua^2^	20
La Flor (Bebedero)	V: MCR^1^, Durango-Coahuila-Chihuahua^2^	17
La Flor (Joyita)	V: MCR^1^, Durango-Coahuila-Chihuahua^2^	17
San Ignacio Yermo	V: MCR^1^, Durango-Coahuila-Chihuahua^2^	11
Laboratory MBR North	V: MCR^1^, Durango-Coahuila-Chihuahua^2^	8
Laboratory MBR South	V: MCR^1^, Durango-Coahuila-Chihuahua^2^	11
La Soledad	V: MCR^1^, Durango-Coahuila-Chihuahua^2^	3
San Carlos	V: MCR^1^, Durango-Coahuila-Chihuahua^2^	5

Roman numerals before the district name correspond to the designation assigned by Bury *et al*. [29].

^1^ MCR = Mapimí Central Region

^**2**^ District V was modified and we included the state of Chihuahua because several sites lie within this region.

## Discussion

An abundance estimate of the Bolson tortoise is key to establishing the conservation status of the species. This is the first range-wide study in which abundance is estimated at the regional scale; thus, it has potentially important implications for further conservation and management actions.

Ecological niche modeling has been a helpful approach for predicting species distributions with conservation purposes, particularly when data are limited [37–41]. Interestingly, niche model outcome probabilities (or similar results) are frequently interpreted as a measure of habitat suitability, thus it is implicitly thought that they somehow provide information about population potential performance [42]; however, this assumption has seldom been tested, mainly due to a lack of information on performance parameters across the whole species range, but when such suitability has been compared with the abundance of species, the results are inconclusive, but generally indicate that this relationship is weak [43–46].

In this study we implemented the Mahalanobis distance to the niche centroid approach to estimate the abundance of the Bolson tortoise across its geographic range [18]. This method is data demanding, which may be a potential pitfall. Several tens of occurrences are needed to obtain a reliable estimation of the species’ niche.

We found a significant relationship suggesting that the abundance of *G*. *flavomarginatus* is strongly determined by the internal structure of the species’ niche throughout its geographic range [20]. Interestingly, we observed that its abundance distribution followed a centralized pattern both in the ecological and geographic spaces, where abundance tends to be highest toward the center of these spaces and decrease toward the boundaries [2,47]. Although irregular and containing unoccupied areas, the shape of the species range is roughly circular, so the centralized pattern found in both ecological and geographic spaces is not surprising [18].

Our results were similar to those reported for the Spotted Turtle (*Clemmys guttata*), in terms of the form of the function and the explanatory power of abundance by the distance to the niche centroid [18]. Interestingly, these authors found that for 9 out of the 10 species analyzed in their study, the relationship between the distance to the centroid and abundance was not inverse, such as in our study, but rather logarithmic or exponential [18]. This has implications in the demography of species, since the reduction in the population size of the chelonids with increasing distance from the niche centroid is abrupt and not, monotonic as in the other species.

On the other hand, the relationship that we found was not as strong as that reported for the White-tailed Deer (*Odocoileus virginianus*; R^2^ = 0.902 and R^2^ = 0.761) [21]. If the hypothesis that the centriod of the ecological niche encompasses the optimal conditions for the species is correct, differences in the predicting power of the model might be due to environmental or ecological variables that were not taken into account [48]. The Bolson tortoise, for instance, is an herbivore that inhabits “sabaneta” (*Pleuraphis mutica*) grassland borders and biotopes with soft slopes, fine-texture soils with a mixture of shrubs (*Larrea divaricata*, *Prosopis juliflora*, *Parthenium incanum*, and *Fluorensia cernua*) and halophilic grasses [26], where cattle also feed. Therefore, the cattle may be interfering with tortoises, directly or indirectly [49]. Studies for other *Gopherus* species have reported that young individuals are frequently kicked or stepped on by cattle [50,51]. Also, the Bolson tortoise is illegally exploited for meat and sold as a pet [26,52,53]; however, there is no hard data to evaluate the impact of these activities on the demography of the species. In addition, the Bolson tortoise has a very low dispersal rate, which may cause low population numbers in suitable habitats, as might be the lack of field surveys in several areas. Therefore, besides abiotic variables, biotic interactions are important factors that also drive species’ distributions [54–56]; and ultimately might influence the species’ abundance [47,57].

The distance to the centroid method is a static approach that does not capture the spatio-temporal dynamics of populations [21]. The method assumes unimodality and centrality of abundance in relation to all environmental variables (i.e., that optimal conditions are always close to the mean values for all variables). This, may not necessarily hold true, because for some variables optimal conditions might actually be closer to the extreme values. Despite these shortcomings, the distance to the centroid approach represented the abundance distribution patterns of the Bolson tortoise fairly well.

Other approaches have been developed to obtain spatial estimates of abundance, like the fractal model [16] and the negative binomial distribution method [17]. This negative binomial distribution approach is the most popular (e.g., Tosh, Reyers & van Jaarsveld [58]; Figueiredo & Grelle [59]), but is strongly dependent on spatial scale [60]. Unfortunately, these methods cannot be directly compared to our implementation of the distance to the niche centroid because their performance is not measured via a determination coefficient. Our approach (i.e., DNC) is more flexible because the results are inferred from presence-absence data, instead of presence-only data as in other methods. Furthermore, for good-fit models the distance to the centroid alone can be a good approximation of the suitability of the environment to the species.

Previous field studies on the abundance of *G*. *flavomarginatus* in some areas of its geographic range reported a marked contrast in tortoise density across its range (5–44 burrows/0.5 km^2^; [29]). Our results suggest that after three decades the abundance of the Bolson tortoise seems to be declining, in some areas by as much as 91%. For instance, Bury et al. [29] reported the presence of 88 individual of this species in Cerros Emilio, in Chihuahua, whereas we only recorded 8 different individuals (Table 4). The largest colonies of tortoise that we found are in the Mapimí Biosphere Reserve, with up to 20 individuals/Km^2^. Previous reports suggest that the abundance of the species has decreased by 25% in the sites for which historical data is available. In 1988 a total of 25 individuals were reported at Las Lolas [29]; but during the two years that our field survey lasted we only observed 18 tortoises at the exact same locality (Table 4). Anecdotal information also suggests that populations seem to be smaller today than a couple of decades ago, particularly at the distributional limits of the species (G. Aguirre, pers. comm.). This trend seems to be a consequence of the expansion of the railroad network, cattle ranching and agriculture in the Chihuahuan Desert during the 20^th^ century [25,33].

**Fig 4:**
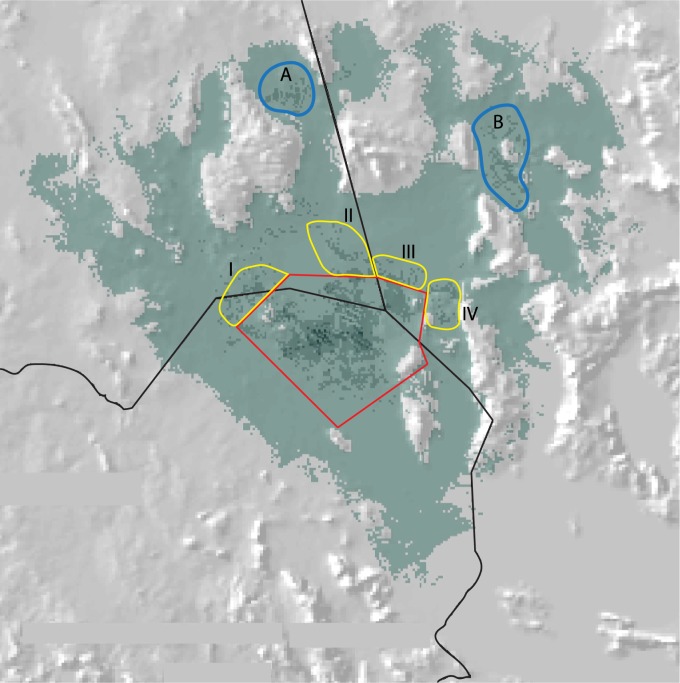
Proposed key sites (yellow polygons) for the conservation of the Bolson tortoise, *Gopherus flavomarginatus*. The red line delineates the current protected polygon of the Mapimí Biosphere Reserve. Sierra del Diablo (A) and Sierra Mojada (B) represent putative high abundance sites; nonetheless, these are low priority areas due to their natural isolation and low human population density.

Based on our results we identified four high-abundance areas, contiguous to the Mapimí Biosphere Reserve polygon, worthy of considering for legal protection (Fig 4). According to our results, the most convenient sites for this purpose are Areas III (236 Km^2^; 26.88°N, -103.59°W) and IV (291 Km^2^; 26.68°N, -103.44°W) in the state of Coahuila because these two areas hold very low human population and road densities, especially Area III. Area I (528 Km^2^; 26.79° N, -104.29°W), and II (575 Km^2^; 27.01° N, -103.98° W) in Chihuahua are the largest, but they are the most populated and intensively used areas for human activities, given its closeness to Lake Palomas. Additionally, Sierra del Diablo (27.63° N, -104.15°W), and Sierra Mojada (27.37° N, -103.11°W), located in the states of Chihuahua and Coahuila respectively, represent important regions in terms of the expected abundance. Currently, these regions are not subjected to any type of protection; however they might be considered of least concern because they are relatively isolated, and human population density is very low (and currently is declining).

Protection of the four areas proposed would add 1,279 Km^2^ to the 3,423.88 Km^2^ currently under protection within the Mapimí Biosphere Reserve polygon for the Bolson tortoise. According to previous studies [61,62], a fifth potential area for the tortoise protection is the Sierra del Diablo District (20 Km^2^ surrounding Cerros Emilio 27.42°N, 103.97°W), in Chihuahua. This area has high levels of abundance according to our model; however, it is located at the northern distribution edge of the species and is similar in area and characteristics to Area IV. Therefore, a carefully thought out strategy would be necessary to create a multi-polygon protected area taking into account the management systems of local land resources. The conservation of the Bolson tortoise is of the highest priority; decisions should be made with the best information available and this study aims to provide relevant information in this regard. The results presented here can be used as a first and simple approach for inferring abundance patterns in space. Such information is important for making management, or conservation decisions for any species.

## Conclusions

This study aims to contribute to the conservation of the Bolson tortoise by providing new insights about the abundance distribution of the species at the range-wide scale. First, the aggregated nature of the distribution of the species across the landscape should be taken into account for conservation strategies. Compared to the other areas where the Bolson tortoise is distributed, the Mapimí Biosphere Reserve successfully protects some of its populations [61] because it holds the largest colonies and actively prohibits their exploitation. Even there, however, the population is still declining. Moreover, given the restricted distribution of the species and the lack of protection in other areas, top priority must be given to including most-if not all- of this species’ range under a protection scheme.
